# Suppression of Tumor Growth and Cell Migration by Indole-Based Benzenesulfonamides and Their Synergistic Effects in Combination with Doxorubicin

**DOI:** 10.3390/ijms23179903

**Published:** 2022-08-31

**Authors:** Phuong Linh Nguyen, Ahmed Elkamhawy, Young Hee Choi, Chang Hoon Lee, Kyeong Lee, Jungsook Cho

**Affiliations:** 1 College of Pharmacy, Dongguk University-Seoul, Goyang 10326, Korea; 2 Department of Pharmaceutical Organic Chemistry, Faculty of Pharmacy, Mansoura University, Mansoura 35516, Egypt

**Keywords:** indole-based benzenesulfonamide, carbonic anhydrase inhibitor, anticancer effect, antimigration, doxorubicin, combination therapy, breast cancer

## Abstract

Pharmacological inhibition of the enzyme activity targeting carbonic anhydrases (CAs) demonstrated antiglaucoma and anticancer effects through pH control. Recently, we reported a series of indole-based benzenesulfonamides as potent CA inhibitors. The present study aimed to evaluate the antitumor effects of these compounds against various cancer cell lines, including breast cancer (MDA-MB-231, MCF-7, and SK-BR-3), lung cancer (A549), and pancreatic cancer (Panc1) cells. Overall, more potent cytotoxicity was observed on MCF-7 and SK-BR-3 cells than on lung or pancreatic cancer cells. Among the 15 compounds tested, **A6** and **A15** exhibited potent cytotoxic and antimigratory activities against MCF-7 and SK-BR-3 cells in the CoCl_2_-induced hypoxic condition. While **A6** and **A15** markedly reduced the viability of control siRNA-treated cells, these compounds could not significantly reduce the viability of CA IX-knockdown cells, suggesting the role of CA IX in their anticancer activities. To assess whether these compounds exerted synergism with a conventional anticancer drug doxorubicin (DOX), the cytotoxic effects of **A6** or **A15** combined with DOX were analyzed using Chou−Talalay and Bliss independence methods. Our data revealed that both **A6** and **A15** significantly enhanced the anticancer activity of DOX. Among the tested pairs, the combination of DOX with **A15** showed the strongest synergism on SK-BR-3 cells. Moreover, this combination further attenuated cell migration compared to the respective drug. Collectively, our results demonstrated that **A6** and **A15** suppressed tumor growth and cell migration of MCF-7 and SK-BR-3 cells through inhibition of CA IX, and the combination of these compounds with DOX exhibited synergistic cytotoxic effects on these breast cancer cells. Therefore, **A6** and **A15** may serve as potential anticancer agents alone or in combination with DOX against breast cancer.

## 1. Introduction

The abnormal and uncontrollable growth of tumor cells often induces the development of strong hypoxic conditions in solid tumors [1,2]. Hypoxia drives metabolic activities of tumor cells from oxidative phosphorylation to aerobic glycolysis, resulting in a high level of lactic acid and accumulation of extracellular H^+^ in the tumor microenvironment [3]. While the increased acidity is toxic to normal cells, tumor cells can adapt to the intracellular pH (pH_i_)/extracellular pH (pH_e_) perturbations by modulating pH-controlling proteins, such as carbonic anhydrase (CA) [3,4]. The activity of CA contributes to the neutralization of the acidic environment by facilitating the reversible hydration of CO_2_ to H^+^ and HCO_3_^−^. To date, 16 different isoforms of CAs have been identified in the human body [5]. Among them, CA IX and XII have been strongly implicated in cancer [5,6]. CA IX is predominantly expressed in many solid tumors. Although CA XII is found in tumor cells, it is also expressed in various organs, such as the eye, reproductive epithelium, and intestine, implicating its role in regulating the normal functioning of these organs [5]. The upregulated CA IX and XII are involved in tumor growth, invasion, metastasis, and drug resistance [7,8,9]. Moreover, high expression of CA IX induced by tumor hypoxia is associated with poor prognosis for a variety of cancers, including invasive breast cancer [10,11,12]. Unlike CA IX, CA XII is not identified as a predictive factor [5]. Considering its exclusive distribution in tumors and critical role in tumor progression, CA IX has emerged as a promising target for cancer therapy.

Over the last decades, numerous studies have been conducted to find potent inhibitors targeting CA IX to develop novel class antitumor agents. Consequently, several promising candidates have been identified [13]. For instance, different classes of sulfonamides and their analogs were found to inhibit CA IX activity and breast cancer cell growth and migration in vitro, and some of them exhibited anticancer activity in xenograft subcutaneous and metastatic mouse models [14,15]. In addition, a series of arylureido-benzenesulfonamides inhibiting CA IX is currently under clinical development to treat solid tumors. A phase Ia clinical trial with a highly selective CA IX inhibitor (SLC-0111) has recently reported a safe and effective outcome in patients with advanced and metastatic solid tumors [16]. This molecule is further progressing to phase Ib/II clinical study.

CA IX is also considered a promising target for adjuvant therapeutic agents to avoid therapeutic failure due to the development of drug resistance or to improve pharmacological efficacy in cancer treatment [17]. Beyond their clinical uses as a monotherapy, additional attention has been paid to CA inhibitors as a potential combination therapy with a conventional cytotoxic agent having a distinct mechanism of action. For example, acetazolamide, a well-known CA inhibitor clinically used to treat glaucoma or edema from heart failure, improved the efficacy and reduced toxicity of a conventional anticancer drug doxorubicin (DOX), presumably by increasing the cellular uptake of DOX in HT29 human colorectal adenocarcinoma cells [18]. In addition, SLC-0111 enhanced the sensitivity of cancer cells to conventional chemotherapies, such as dacarbazine and temozolomide in melanoma cells, DOX in breast cancer cells, and 5-fluorouracil in colon cancer cells [19]. Given the growing evidence, it is worth advocating the use of CA inhibitors in combination with conventional anticancer drugs to improve the efficacy of cancer treatment.

**Figure 1:**
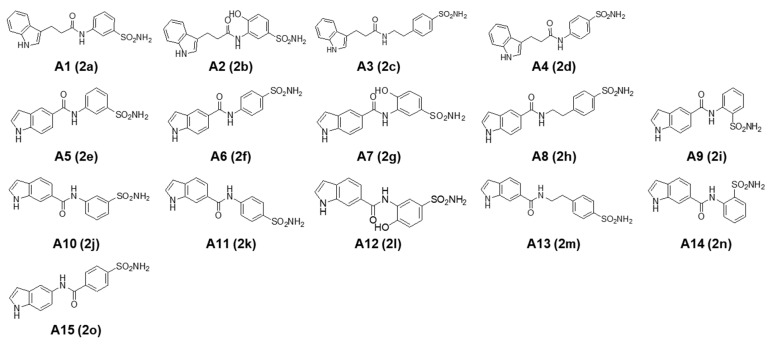
Chemical structures of indole-based benzenesulfonamides (**A1−A15**). The compound codes (**2a−2o**) reported to be potent carbonic anhydrase inhibitors [20] are also provided in parentheses.

A series of novel indole-based benzenesulfonamides were synthesized and biologically evaluated as potential CA inhibitors by measuring the inhibitory constant (*K_i_*) against four human CA isoforms (CA I, II, IX, and XII). Among these derivatives, compounds **2a−d**, **2f**, **2h**, and **2o** were reported to exhibit potent and selective inhibition of the CA II isoform, with compound **2a** being the most potent [20]. In the present study, these compounds (**2a−2o**) were sequentially renamed (**A1−A15**, Figure 1) and evaluated for their anticancer activities against various cancer cell lines. Three types of breast cancer cells with distinct characteristics of specifically targeted receptors, including triple-negative MDA-MB-231, estrogen receptor (ER)^+^/human epidermal growth factor receptor 2 (HER2)^−^ MCF-7, and ER^−^/HER2^+^ SK-BR-3 cells, are employed along with lung cancer cells (A549) and pancreatic cancer cells (Panc1). We also investigated the functional roles of CA isoforms in the anticancer activities of these compounds. To test whether the efficacy could be synergistically improved, we further assessed the cytotoxic effects of the most potent compounds (**A6** and **A15**) in combination with DOX, using Chou−Talalay and Bliss independence methods. Based on our findings, we demonstrated in this study that **A6** (*N*-(4-sulfamoylphenyl)-1H-indole-5-carboxamide) and **A15** (*N*-(1H-indol-5-yl)-4-sulfamoylbenzamide) suppressed tumor growth and cell migration of MCF-7 and SK-BR-3 cells through inhibition of CA IX, and the combination of these compounds with DOX exhibited synergistic cytotoxic effects on these breast cancer cells.

## 2. Results

### 2.1. Cytotoxic Effects of Indole-Based Benzenesulfonamide Derivatives on Various Types of Cancer Cells

#### 2.1.1. Preliminary Screening for Their Cytotoxic Effects on Cancer Cells

Fifteen compounds with indole-based benzenesulfonamides (**A1−A15**) were screened for their cytotoxic effects against various human cancer cell lines, including breast cancer cells (MDA-MB-231, MCF-7, and SK-BR-3 cells), lung cancer cells (A549), and pancreatic cancer cells (Panc1). Cobalt (II) chloride (CoCl_2_) mimics hypoxia in vitro by stabilizing hypoxia-inducible factor-alpha (HIF-1α) [21,22]. In this study, CoCl_2_ (100 μM) was used to establish a chemically-induced hypoxic condition. For preliminary screening, cancer cells were treated with the test compounds at 10 and 100 µM for 48 h in the presence of CoCl_2_, and the cell viability was measured by the MTT assay.

**Figure 2:**
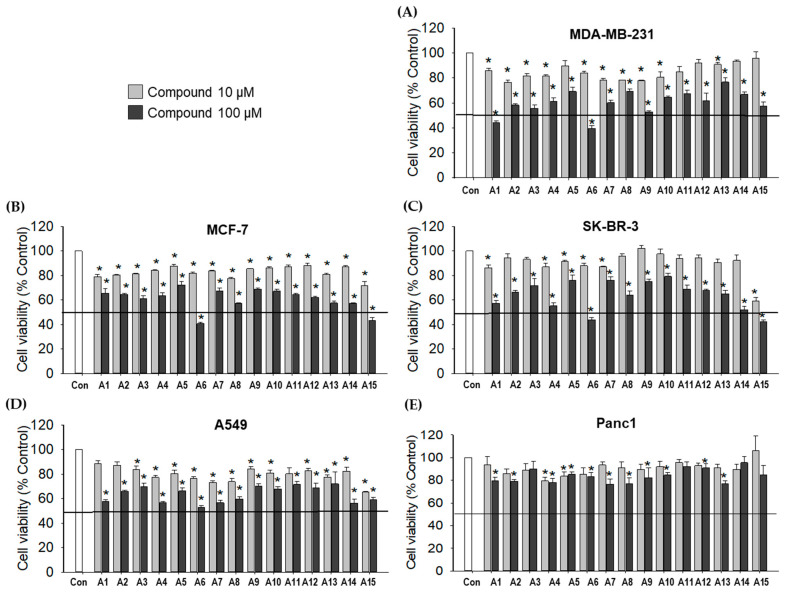
Effects of indole-based benzenesulfonamide derivatives on the viability of cancer cells. (**A**) MDA-MB-231, (**B**) MCF-7, (**C**) SK-BR-3, (**D**) A549, and (**E**) Panc1 cells were treated with the test compounds (10 and 100 μM) for 48 h under CoCl_2_-induced hypoxic condition. Control cells were treated with the media containing 0.2% dimethyl sulfoxide (DMSO) instead. Cell viability was determined by MTT assay as described in the Materials and Methods. The viability of vehicle (DMSO)-treated control cells was defined as 100%, and the viability of the cells treated with test compounds was expressed as a percentage of the control measured in the vehicle-treated cells in each cell line. Data are displayed as mean ± SEM from three independent experiments. * *p* < 0.05 vs. the vehicle-treated control cells. Con, control.

Our screening tests indicated that most compounds exhibited significant cytotoxicity in five types of cancer cells at the concentration of 100 μM, although more potent cytotoxicity was observed in breast cancer cell lines than lung or pancreatic cancer cells (Figure 2). Among the breast cancer cells, MDA-MB-231 showed higher sensitivity to **A1** and **A6** (Figure 2A), while MCF-7 and SK-BR-3 cells were more sensitive to **A6** and **A15**, inhibiting the cell viability by more than 50% (Figure 2B,C). Based on these findings, compounds **A1**, **A6**, and **A15** were chosen to determine their concentrations inhibiting 50% cell viability (IC_50_) in breast cancer cells.

#### 2.1.2. Determination of IC_50_ Values of the Selected Compounds to Inhibit Viability of Breast Cancer Cells

To determine the IC_50_ values of the selected compounds in breast cancer cells, the concentration-dependent cytotoxic effects of **A1** and **A6** on MDA-MB-231 cells and **A6** and **A15** on MCF-7 and SK-BR-3 cells were assessed by the MTT assay at 48 h of treatment. The cytotoxic effects of these compounds were compared with that of SLC-0111, a potent CA inhibitor particularly targeting CA IX and XII [23].

**Figure 3:**
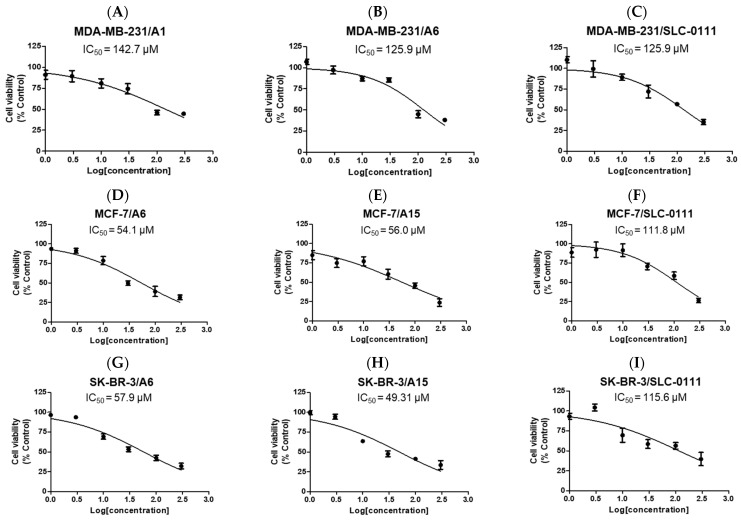
Concentration-dependent effects of the selected compounds on the viability of breast cancer cells. (**A**─**C**) Effects of **A1**, **A6**, and SLC-0111 on the viability of MDA-MB-231 cells. (**D─F**) Effects of **A6**, **A15**, and SLC-0111 on the viability of MCF-7 cells. (**G─I**) Effects of **A6**, **A15**, and SLC-0111 on the viability of SK-BR-3 cells. Cells were treated with the indicated compounds at 1, 3, 10, 30, 100, and 300 μM for 48 h under CoCl_2_-induced hypoxic condition. Control cells were treated with the media containing 0.6% DMSO instead. Cell viability was determined by MTT assay as described in the Materials and Methods. The viability of vehicle (DMSO)-treated control cells was defined as 100%, and the cell viability was expressed as a percentage of the control measured in the vehicle-treated cells in each cell line. Data are displayed as mean ± SEM from three independent experiments. IC_50_ values were calculated using GraphPad Prism, as described in the Materials and Methods.

Overall, the selected compounds exhibited similar cytotoxic patterns in each cell type (Figure 3). While the IC_50_ values of **A1** and **A6** on MDA-MB-231 cells were determined to be over 100 µM (Figure 3A,B), those of **A6** and **A15** on MCF-7 (Figure 3D,E) and SK-BR-3 (Figure 3G,H) cells were close to 50 μM. These data also indicate that MCF-7 and SK-BR-3 cells are more sensitive than MDA-MB-231 cells to these compounds. The potencies of **A1** and **A6** on MDA-MB-231 cells were comparable to that of SLC-0111 (Figure 3C). However, **A6** and **A15** exhibited more potent inhibition than SLC-0111 on MCF-7 and SK-BR-3 cells (Figure 3F,I, respectively). Thus, **A6** and **A15** were selected for further investigation in MCF-7 and SK-BR-3 cells in this study.

### 2.2. Involvement of CA IX Isoform in **A6**- and **A15**-Induced Cytotoxic Effects on MCF-7 and SK-BR-3 Cells

#### 2.2.1. Protein Expression of CA I, II, IX, and XII Isoforms in CoCl_2_-Treated MCF-7 and SK-BR-3 Cells

**Figure 4:**
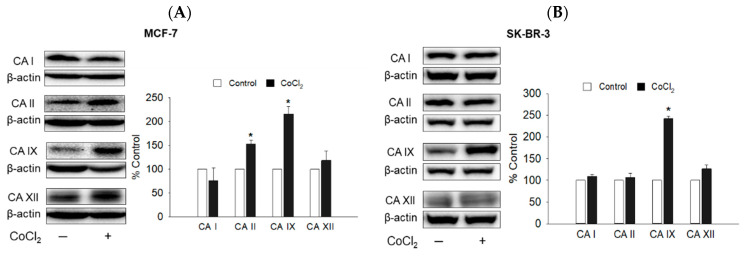
Protein expression of CA I, II, IX, and XII isoforms in MCF-7 and SK-BR-3 cells. (**A**) MCF-7 and (**B**) SK-BR-3 cells were exposed to vehicle or CoCl_2_ (100 µM) for 48 h. Protein levels of CA isoforms were determined by western blotting analyses using anti-CA I, II, IX, and XII antibodies, respectively. β-Actin was used for normalization. The expression level of each isoform in CoCl_2_-treated cells (black bar) is expressed as a percentage of the respective control measured in the vehicle (DMSO)-treated cells without CoCl_2_ (white bar). Representative blots are shown. Data are displayed as mean ± SEM from three independent experiments. * *p* < 0.05 vs. the vehicle-treated control cells.

The indole-based benzenesulfonamides were originally designed and synthesized as potential human CA inhibitors [20]. Prior to the identification of CA isoform(s) mediating the cytotoxic effects of **A6** and **A15**, we first examined the protein levels of several CA isoforms, including ubiquitously expressed cytosolic CA I and II and cancer-associated transmembrane CA IX and XII, in the presence or absence of CoCl_2_ in MCF-7 and SK-BR-3 cells using western blotting. In keeping with previous findings in breast cancer cells [15,24], the expression of the CA IX isoform was markedly upregulated in both cells by the CoCl_2_-induced hypoxic condition, compared to that of the normoxic control condition (Figure 4). By contrast, the protein levels of CA I, II, and XII were not significantly altered by CoCl_2_ treatment, except for CA II in MCF-7 cells. Based on these results, we next examined the effects of **A6** and **A15** on the CoCl_2_-induced CA IX expression in MCF-7 and SK-BR-3 cells.

#### 2.2.2. Suppression of CoCl_2_-Induced CA IX Expression by **A6** and **A15**

**Figure 5:**
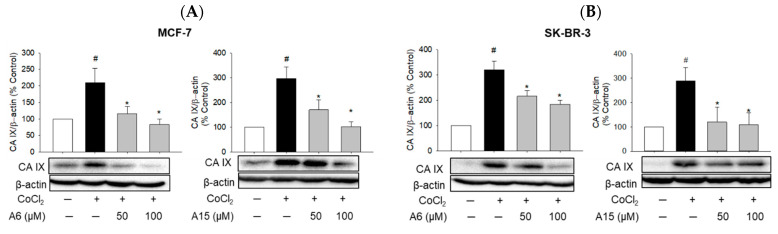
Suppression of CoCl_2_-induced CA IX expression by **A6** and **A15** in MCF-7 and SK-BR-3 cells. (**A**) MCF-7 and (**B**) SK-BR-3 cells were treated with **A6** or **A15** (50 and 100 µM) in the presence or absence of CoCl_2_ (100 µM) for 48 h. Control cells were treated with the media containing 0.1% DMSO instead. Protein levels of CA IX were determined by western blotting analyses using anti-CA IX antibody. β-Actin was used for normalization. The expression level of CA IX is expressed as a percentage of the control measured in the vehicle-treated control cells. Representative blots are shown. Data are displayed as mean ± SEM from three independent experiments. # *p* < 0.05 and * *p* < 0.05 vs. the vehicle-treated control cells and CoCl_2_-treated cells, respectively.

CA IX has been identified as a poor prognostic biomarker for distant metastases of cancer and overall survival, and pharmacological inhibition of CA IX impairs tumor growth and cell migration [25,26]. To test the effects of **A6** and **A15** on the CA IX expression in MCF-7 and SK-BR-3 cells, the cells were treated with **A6** or **A15** at 50 and 100 µM in the presence or absence of CoCl_2_ for 48 h. Our western blotting analyses showed that both **A6** and **A15** explicitly suppressed the CoCl_2_-induced expression of the CA IX isoform in MCF-7 and SK-BR-3 cells (Figure 5).

#### 2.2.3. Cytotoxic Effects of **A6** and **A15** on CA IX-Knockdown MCF-7 and SK-BR-3 Cells

**Figure 6:**
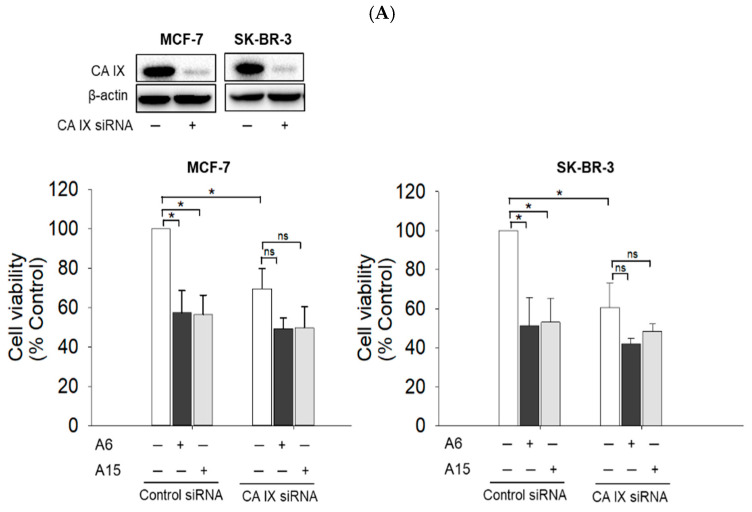

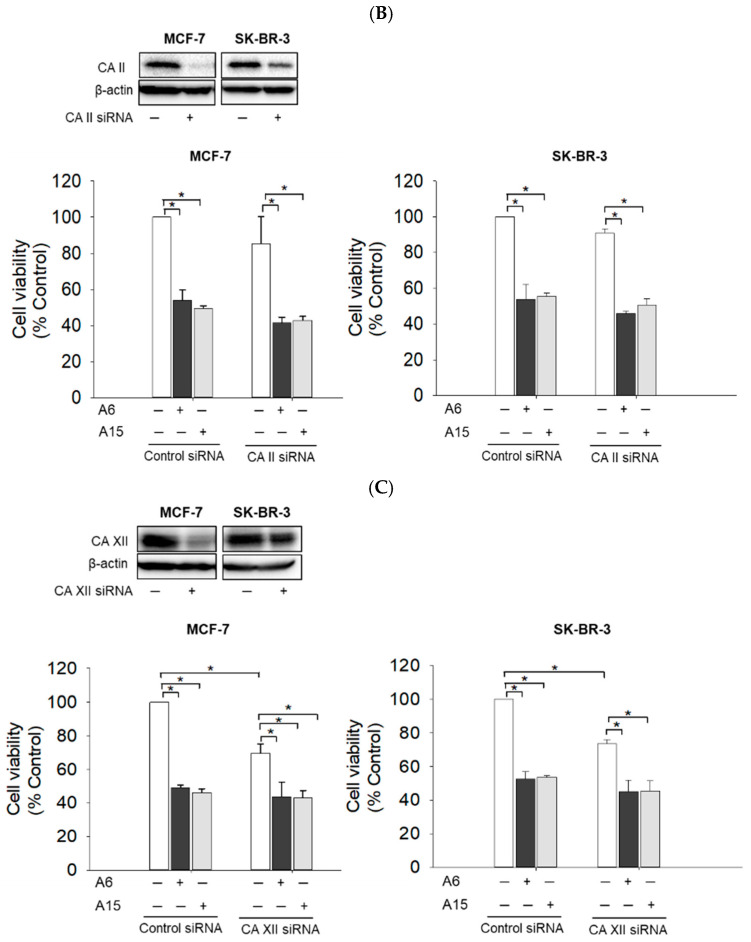
Effects of **A6** and **A15** on the viability of MCF-7 and SK-BR-3 cells transfected with siRNA targeting CA IX, II, or XII. MCF-7 and SK-BR-3 cells were transfected with control siRNA or siRNA targeting (**A**) CA IX, (**B**) CA II, and (**C**) CA XII. Western blotting analyses were performed as described in the Materials and Methods to confirm the efficiency of the respective knockdown. Representative blots are shown. Transfected cells were exposed to **A6** or **A15** at 50 µM for 48 h. Control cells were treated with the media containing 0.1% DMSO instead. Cell viability was determined by MTT assay as described in the Materials and Methods and expressed as a percentage of the control measured in the vehicle (DMSO)-treated cells transfected with control siRNA. Data are displayed as mean ± SEM from at least three independent experiments. *p < 0.05 vs. the vehicle-treated control cells transfected with control siRNA. ns, not significant.

To investigate the role of CA IX in the cytotoxic effects of **A6** and **A15**, MCF-7 and SK-BR-3 cells were transfected with small interfering RNA (siRNA) targeting CA IX, treated with our compounds (50 µM) for 48 h, and then the cell viability was measured by the MTT assay. As shown in Figure 6A, the CA IX knockdown significantly reduced the cell viability by approximately 30~40% in both cells, suggesting the crucial role of CA IX in tumor cell survival. While **A6** and **A15** markedly reduced the viability of control siRNA-treated cells, these compounds could not significantly reduce the viability of CA IX-knockdown cells, suggesting the role of CA IX in their anticancer activities.

As previously described (Figure 4A), CoCl_2_ significantly induced CA II expression in MCF-7 cells. Moreover, compounds **A6** and **A15** were reported to potently inhibit the CA II isoform, with *K_i_* values of 16.0 and 7.5 nM, respectively [20]. These findings prompted us to examine whether CA II played any role in the cytotoxic effects of **A6** and **A15** on MCF-7 and SK-BR-3 cells. We found that the viability of CA II-knockdown cells was not significantly different from that of the control siRNA-treated cells (Figure 6B), suggesting that CA II expression was not critical for the survival of these cells. Unlike CA IX-knockdown cells, the viability of CA II-knockdown cells was significantly reduced by **A6** or **A15** to a level similar to that of the control siRNA-treated cells (Figure 6B). Given that knockdown of the CA II isoform did not affect the **A6**- or **A15**-induced cytotoxicity on both cell types, CA II appeared not to participate in the cytotoxic effects of these compounds.

In addition to CA IX, CA XII is also implicated in tumor progression, promoting tumor growth and metastases formation [5]. Although the CoCl_2_-induced hypoxic condition did not upregulate CA XII expression in breast cancer cells (Figure 4), we further tested whether this isoform mediated the cytotoxic effects of **A6** and **A15**. As shown in Figure 6C, the viability of CA XII-knockdown cells was significantly reduced compared to that of control siRNA-treated cells, strengthening its role in tumor cell survival. However, similar to CA II knockdown, the viability of CA XII-knockdown cells was further reduced by **A6** or **A15** (Figure 6C), indicating that CA XII did not mediate the cytotoxic effects of **A6** and **A15** on these breast cancer cells. Taken together, these results demonstrate that **A6** and **A15** exert cytotoxic effects by inhibiting CA IX in MCF-7 and SK-BR-3 cells.

### 2.3. Effects of **A6** and **A15** on the Migration of MCF-7 and SK-BR-3 Cells

The regulation of pH by CA IX plays a crucial role in tumor cell migration [27]. To investigate the effects of **A6** and **A15** on MCF-7 and SK-BR-3 cell migration, the cells were treated with the test compounds or SLC-0111 at 50 μM for 24 and 48 h, and the cell migration was assessed by the wound healing assay.

**Figure 7:**
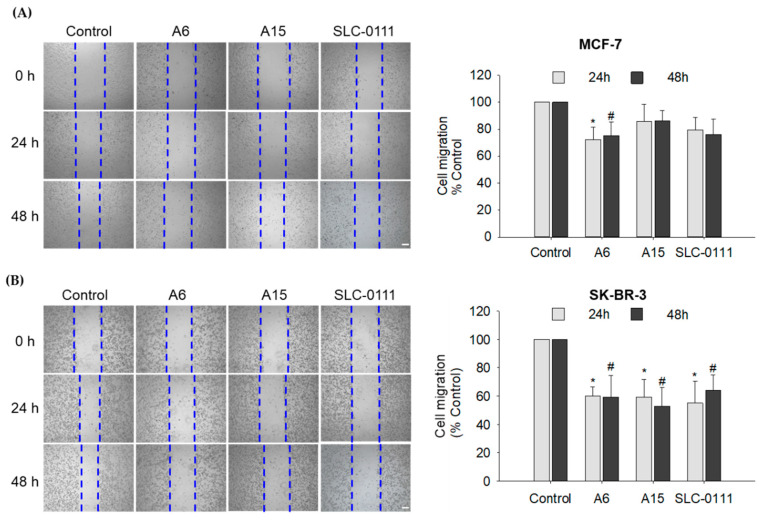
Effects of **A6** and **A15** on the migration of MCF-7 and SK-BR-3 cells. (**A**) MCF-7 and (**B**) SK-BR-3 cells were treated with **A6**, **A15**, or SLC-0111 at 50 μM for 24 or 48 h. Control cells were treated with serum-free media containing 0.1% DMSO instead. Cell migration was determined by wound healing assay, as described in the Materials and Methods. Representative images are shown. Data are displayed as mean ± SEM from three independent experiments. * *p* < 0.05 and # *p* < 0.05 vs. the vehicle-treated control cells for 24 and 48 h, respectively. Scale bar, 250 µm.

In MCF-7 cells, **A6** significantly attenuated the cell migration at 24 and 48 h, while **A15** and SLC-0111 showed no effect (Figure 7A). In SK-BR-3 cells, both **A6** and **A15** reduced the cell migration (Figure 7B), which was more effective than in MCF-7 cells (Figure 7A,B). The inhibition of SK-BR-3 cell migration by **A6** or **A15** was comparable to that by SLC-0111. Collectively, our data demonstrate that, while the migration of MCF-7 cells was inhibited by **A6** only, SK-BR-3 cell migration was inhibited by both **A6** and **A15**.

### 2.4. Cytotoxic Effects of **A6** or **A15** in Combination with DOX on MCF-7 and SK-BR-3 Cells

#### 2.4.1. Cytotoxic Effect of DOX as a Single Treatment

**Figure 8:**
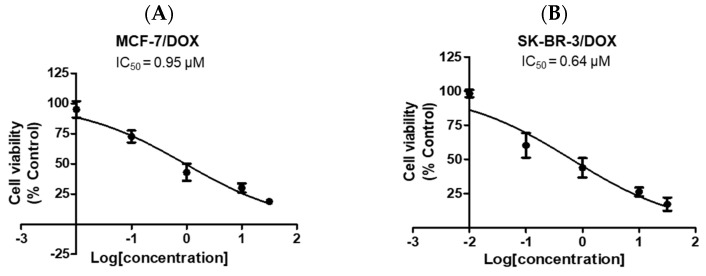
Effects of doxorubicin (DOX) on the viability of MCF-7 and SK-BR-3 cells. (**A**) MCF-7 and (**B**) SK-BR-3 cells were treated with DOX at 0.01, 0.1, 1, 10, and 30 μM. Control cells were treated with the media containing 0.03% DMSO instead. Cell viability was determined by MTT assay as described in the Materials and Methods. Data are displayed as mean ± SEM from three independent experiments. IC_50_ values were calculated using GraphPad Prism, as described in the Materials and Methods.

DOX is a conventional anticancer agent typically administered in conjunction with other chemotherapeutic drugs for the management of advanced-stage breast cancer [19]. To determine the DOX concentration for combination treatment with **A6** or **A15**, we first evaluated the cytotoxic effect of DOX on MCF-7 and SK-BR-3 cells and calculated its IC_50_ values. The cells were treated with DOX at 0.01, 0.1, 1, 10, and 30 μM for 48 h in CoCl_2_-induced hypoxic conditions, and the cell viability was evaluated by MTT assay. DOX concentration-dependently suppressed the viability of MCF-7 (Figure 8A) and SK-BR-3 (Figure 8B) cells and yielded IC_50_ values of 0.95 and 0.64 μM, respectively, in these cells.

#### 2.4.2. Cytotoxic Effects of DOX in Combination with **A6** or **A15**

**Figure 9:**
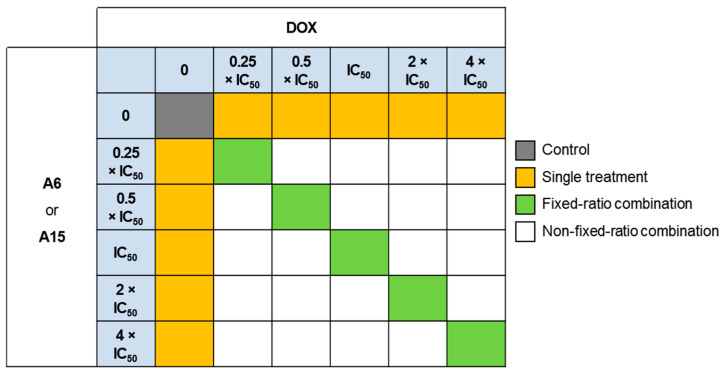
Concentrations of doxorubicin (DOX), **A6**, **A15**, and DOX combined with **A6** or **A15** at fixed ratios tested in MCF-7 and SK-BR-3 cells. Light blue boxes represent the fixed concentrations of each test compound; dark gray box represents the vehicle-treated control; yellow boxes represent the concentrations of **A6**, **A15**, or DOX for single treatment; green boxes represent the concentrations of fixed-ratio combinations; white boxes represent the concentrations of non-fixed-ratio combinations.

To assess the cytotoxic effects of DOX in combination with **A6** or **A15** on MCF-7 and SK-BR-3 cells, the cells were treated with DOX, **A6**, **A15**, or DOX combined with **A6** or **A15** for 48 h at the concentrations indicated (Figure 9). As reported previously [28,29,30], the concentrations of each compound were selected as multiples of the respective IC_50_ value, including 0.25 × IC_50_, 0.5 × IC_50_, IC_50_, 2 × IC_50_, and 4 × IC_50_. Based on our earlier determinations (Figure 3 and Figure 8), the IC_50_ values of **A6** and **A15** were set at 50 μM in both cells, and the IC_50_ values of DOX in MCF-7 and SK-BR-3 cells were set at 1.0 and 0.5 μM, respectively.

**Figure 10:**
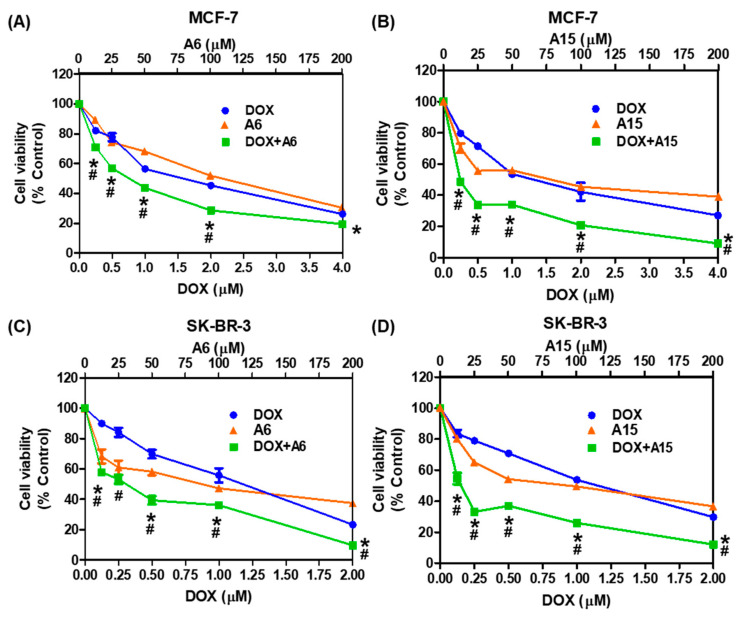
Cytotoxic effects of **A6** or **A15** alone or in combination with DOX on MCF-7 and SK-BR-3 cells. (**A**,**B**) MCF-7 and (**C**,**D**) SK-BR-3 cells were exposed to the indicated concentrations of **A6** or **A15** alone or in combination with DOX at fixed ratios for 48 h, as illustrated in Figure 9. Control cells were treated with the media containing 0.4% DMSO instead. The IC_50_ values of **A6** and **A15** in both cell types, DOX in MCF-7 cells, and DOX in SK-BR-3 cells are set at 50, 1.0, and 0.5 μM, respectively. Cell viability was determined by MTT assay. Data are displayed as mean ± SEM from three independent experiments. # *p* < 0.05 and * *p* < 0.05 vs. DOX-treated cells and **A6**- or **A15**-treated cells, respectively.

As shown in Figure 10, DOX in combination with **A6** or **A15** exhibited stronger inhibition of cell viability than the respective individual treatment in both MCF-7 and SK-BR-3 cells. In MCF-7 cells, for example, the combination at 2 × IC_50_ of DOX (i.e., 2 μM) with **A6** (i.e., 100 μM) significantly enhanced the cytotoxicity, exhibiting approximately 30% cell viability of the control (Figure 10A). Similarly, the combined treatment of MCF-7 cells with DOX and **A15** caused marked cytotoxicity at all combinations tested in this study (Figure 10B). In SK-BR-3 cells, all combinations of DOX with **A6** or **A15** exhibited greatly enhanced inhibition of cell viability (Figure 10C,D). The combination treatment at the highest concentration (4 × IC_50_) of DOX and **A6** or **A15** exhibited the most effective cytotoxicity on both cell types, resulting in less than 20% cell viability. The cytotoxicity induced by the single treatment was significantly lower than that of the combined treatment.

#### 2.4.3. Synergistic Effects of **A6** or **A15** in Combination with DOX on Cell Viability: Evaluation by Chou−Talalay Method

Combination therapy is widely used for the treatment of many intractable or refractory diseases, such as cancer, mainly to achieve synergistic efficacy or avoid drug resistance [31]. By definition, synergism is more than an additive effect of the combined drugs, while antagonism is less than an additive effect. To quantitatively determine whether the combined treatment of DOX with **A6** or **A15** exerted synergism, additivity, or antagonism, Chou−Talalay and Bliss independence methods were used to analyze our data in this study.

**Figure 11:**
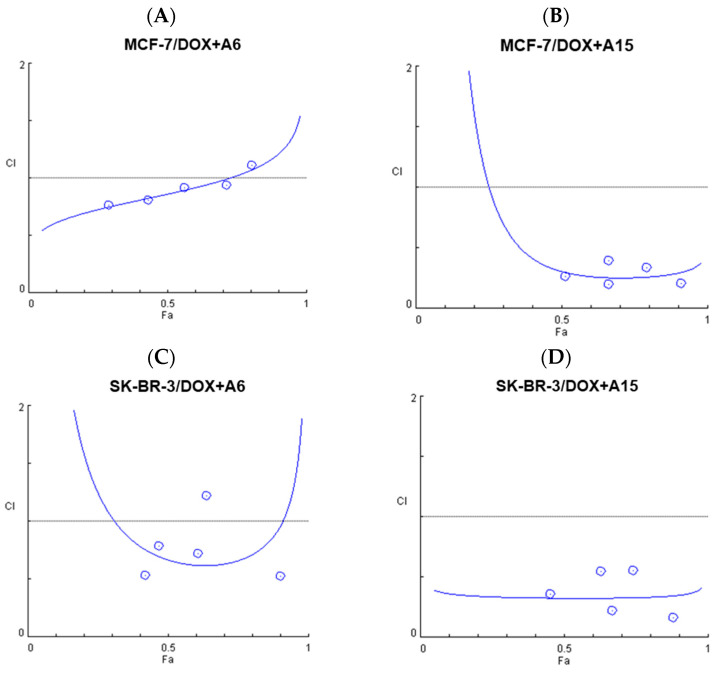
Fractional effect (*F_a_*) and combination index (CI) plots obtained from CompuSyn Report for the combinations of doxorubicin (DOX) with **A6** or **A15** on MCF-7 and SK-BR-3 cells. (**A**,**B**) DOX and (**A**) **A6** or (**B**) **A15** on MCF-7 cells; (**C**,**D**) DOX and (**C**) **A6** or (**D**) **A15** on SK-BR-3 cells. CI is plotted on the Y-axis as a function of the effect level (*F_a_*) on the X-axis to validate synergism of the combination pairs.

The cytotoxic effects measured at the fixed-ratio combination treatments (as illustrated in Figure 9) were analyzed using CompuSyn software, according to the Chou−Talalay method as reported previously [28,32]. This method provides the computerized estimation of combination index (CI) values, defined as the sum of the ratios between combinational and individual concentrations to achieve a specific activity [28]. The CI values are used for quantitative classification of synergism (CI < 1), additivity (CI = 1), and antagonism (CI > 1) at the concentrations tested [28,31]. The CI values and the fractional effects (*F_a_*) of the combination treatment of DOX with **A6** or **A15** at fixed ratios on MCF-7 and SK-BR-3 cells and the plots of *F_a_* and CI are shown in Table 1 and Figure 11, respectively. *F_a_* is indicated by a value from 0 to 1, where 0 signifies that the test compound(s) does not affect the cell viability, and 1 signifies that it produces a full cytotoxic effect.

The combinations of DOX with **A6** or **A15** resulted in synergistic effects in almost all pairs tested on both cell types (Table 1 and Figure 11). The CI values below 1 representing synergism are highlighted in red in Table 1. The combinations of DOX with **A6** acted synergistically on MCF-7 cells at all pairs of concentrations tested, except the pair of 4 × IC_50_ of each drug (Table 1). The synergistic effect of DOX combined with **A6** decreased gradually with increasing concentration, reaching antagonism (CI > 1) at the concentration of 4 × IC_50_ of each compound (Table 1 and Figure 11A). By contrast, the combination of DOX with **A15** on MCF-7 cells showed promising results, with all pairs displaying synergism (CI < 1) and high *F_a_* values (> 0.5) (Table 1 and Figure 11B). Particularly, the pair of 4 × IC_50_ of each compound reached an *F_a_* value of 0.9 (Table 1).

On SK-BR-3 cells, the synergistic effect of DOX and **A6** exhibited a decreasing tendency with increasing concentration up to 2 × IC_50_ of each compound (Table 1 and Figure 11C). However, the synergism returned with the pair of 4 × IC_50_ of each compound, which produced an *F_a_* of 0.9 and CI below 1 (Table 1). Although a similar tendency was observed with the combination of DOX with **A15** on SK-BR-3 cells, all pairs tested in this study exhibited synergistic effects with a maximum *F_a_* of 0.88 (Table 1 and Figure 11D). The synergistic pair with the lowest concentrations (0.125 µM DOX and 12.5 µM **A15**) was chosen for further investigation of the antimigratory effect. Synergistic effects of the combination pairs of DOX and **A6** or **A15** on the viability of MCF-7 and SK-BR-3 cells were further analyzed by the Bliss independence method.

#### 2.4.4. Synergistic Effects of **A6** or **A15** in Combination with DOX on Cell Viability: Evaluation by Bliss Independence Method

In addition to the Chou−Talalay method, the Bliss independence method was also used to calculate the expected responses of the combination treatment of DOX with **A6** or **A15** using SynergyFinder 2.0 software [33]. SynergyFinder is a web application for interactive analysis and visualization of two or more drug combination screening data. The Bliss independence analysis implies a stochastic process in which two drugs produce independent effects, and the expected combination effect is estimated based on the probability of independent events.

**Figure 12:**
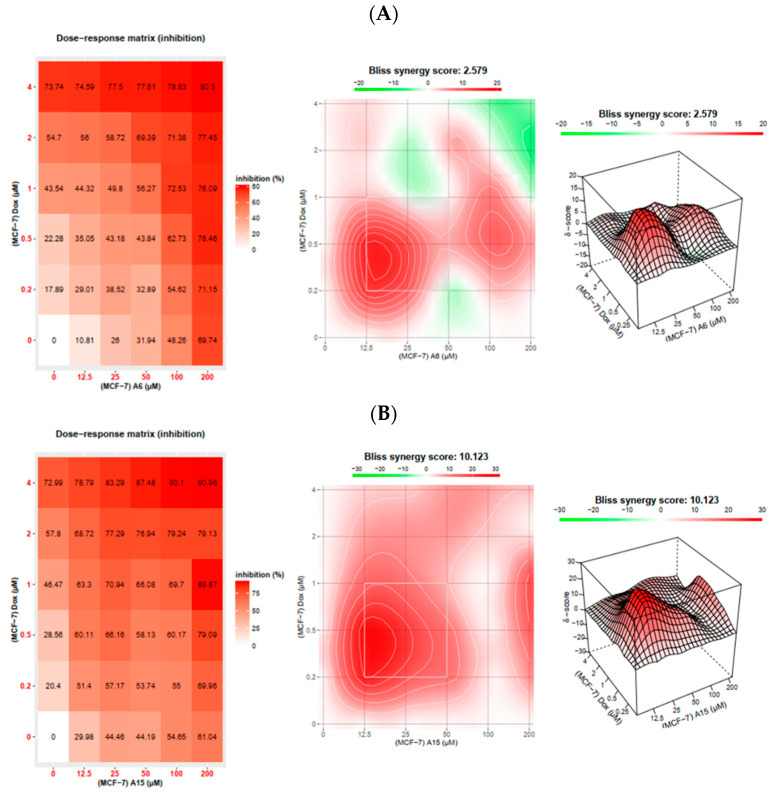

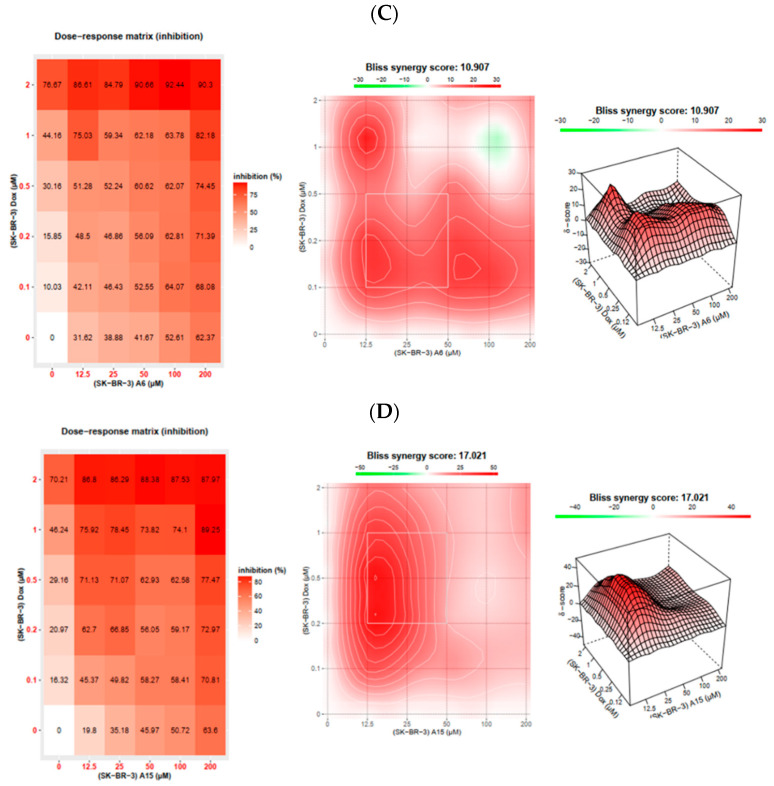
Dose−response matrix inhibition for the combination treatment of doxorubicin (DOX) with **A6** or **A15** on MCF-7 and SK-BR-3 cells and their 2D and 3D synergy maps. (**A**,**B**) DOX and (**A**) **A6** or (**B**) **A15** on MCF-7 cells; (**C**,**D**) DOX and (**C**) **A6** or (**D**) **A15** on SK-BR-3 cells. Synergy maps are highlighted in red and green colors for the synergistic and antagonistic regions, respectively. Bliss independence method was applied to calculate the expected responses of the combination treatment using SynergyFinder 2.0 software, as described in the Materials and Methods.

The MCF-7 and SK-BR-3 cells were treated with DOX, **A6**, **A15**, or DOX combined with **A6** or **A15** at fixed or non-fixed drug combinations, as designed in Figure 9. The *F_a_* effects calculated from the MTT assay were imported to the software along with the drug concentrations. The synergy score for a drug combination is averaged over all dose combination measurements. The calculated synergy scores below −10, from −10 to 10, and above 10 indicate antagonism, additivity, and synergism, respectively [33]. Results for synergistic combinations, including the dose−response matrix inhibition and the two- and three-dimensional (2D and 3D) synergy maps, are provided in Figure 12.

As shown in Figure 12A, the combination of DOX with **A6** in MCF-7 cells obtained an average synergy score of 2.6, indicating an additive effect. According to the synergy maps, the synergistic area was positioned at the pairs with the lower concentrations of each agent (0.2~0.5 μM DOX and 12.5~25 μM **A6**, corresponding to 0.25 × IC_50_−0.5 × IC_50_, respectively) (Figure 12A). The combination of DOX with **A15** in MCF-7 cells (Figure 12B) and the combination of DOX with **A6** in SK-BR-3 cells (Figure 12C) demonstrated synergism in the Bliss model, with the synergy scores of more than 10. In the respective synergy map, the synergistic areas were also indicated at the concentrations of 0.25 × IC_50_−0.5 × IC_50_ (Figure 12B,C). In parallel with the findings from the Chou−Talalay analysis, the highest synergy score was observed with the combination of DOX with **A15** in SK-BR-3 cells, with a synergy score of approximately 17 (Figure 12D). In this combination, the synergistic area was located between the DOX concentrations of 0.2−0.5 µM (0.5 × IC_50_−1 × IC_50_) and **A15** concentrations of 12.5−25 µM (0.25 × IC_50_−0.5 × IC_50_) (Figure 12D). Furthermore, green highlights representing antagonistic areas are not seen in the 2D and 3D synergy maps for DOX and **A15** combination in SK-BR-3 cells (Figure 12D), confirming the synergistic effects of all combination pairs.

Taken together, in agreement with the Chou−Talalay analysis, the Bliss independence model also proved the overall synergism in all combination pairs tested in both types of breast cancer cells, except for the combination of DOX with **A6** in MCF-7 cells. Therefore, both methods demonstrated synergistic cytotoxic effects of **A6** or **A15** in combination with DOX on breast cancer cells, with the most promising synergism displayed by **A15** and DOX in SK-BR-3 cells.

### 2.5. Effects of **A6** or **A15** in Combination with DOX on Cell Migration of SK-BR-3 Cells

As presented in Figure 7, our wound healing data suggested that SK-BR-3 cells were more sensitive than MCF-7 cells to **A6** and **A15** because these compounds inhibited the SK-BR-3 cell migration more effectively than the migration of MCF-7 cells. Hence, to confirm the synergistic effects of the combination treatment of DOX with **A6** or **A15** on cell migration, we investigated their effects on SK-BR-3 cells. Based on the results from the Chou−Talalay method (Table 1), the synergistic pair of 0.125 µM DOX and 12.5 µM **A15** (or **A6**), the concentrations corresponding to 0.25 × IC_50_ of each, was chosen to examine their synergistic effects on SK-BR-3 cell migration.

**Figure 13:**
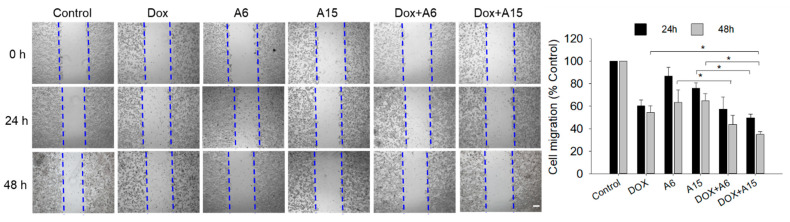
Synergistic effects of the combination treatment of doxorubicin (DOX) with **A6** or **A15** on the migration of SK-BR-3 cells. Cells were exposed to **A6** (12.5 μM), **A15** (12.5 μM), DOX (0.125 μM), or their combinations for 24 or 48 h, as indicated. Control cells were treated with serum-free media containing 0.025% DMSO instead. Alterations in cell migration were measured at 24 and 48 h by wound healing assay, as described in the Materials and Methods. Representative images are shown. Data are displayed as mean ± SEM from three independent experiments. * *p* < 0.05. Scale bar, 250 µm.

The cell migration in the presence of **A6** or **A15** was reduced to approximately 60% of the vehicle-treated control cells during 48 h (Figure 13). When the cells were simultaneously treated with **A6** or **A15** and DOX for 48 h, the antimigratory effects were significantly enhanced compared to **A6** or **A15** alone, further inhibiting the cell migration by up to 30−40% of the control. Moreover, the enhanced antimigratory effect of the combined treatment of DOX with **A15** differed significantly from that of DOX alone (Figure 13). While the effect of DOX and **A6** was not significantly different from that of **A6** alone at 24 h, the antimigratory effect of DOX and **A15** was enhanced significantly at 24 h compared to **A15** alone. Taken together, the combined treatment with **A6** or **A15** and DOX at the concentration pairs of 0.25 × IC_50_ of each synergistically enhanced the inhibition of SK-BR-3 cell migration.

## 3. Discussion

CAs play key roles in tumor progression, promoting tumor cell proliferation and metastasis, and drug resistance through the regulation of pH [6,7,15,34,35]. In particular, the CA IX isoform is induced in hypoxic conditions of cancer and enhances the production of extracellular H^+^ and HCO_3_^─^. While HCO_3_^─^ is shuttled into the cytoplasm to buffer the pH_i_, H^+^ contributes to the acidification of the extracellular medium, which, in turn, promotes extracellular matrix collapse and tumor cell migration and invasion. Accordingly, aggressive tumor cells may survive in the hostile environment imposed by hypoxia. Pharmacological inhibition of CAs, particularly targeting CA IX, has been implicated in inhibiting tumor cell survival and reducing invasiveness [25,36]. CoCl_2_ is most widely used as a chemical inducer of hypoxia, which enables in vitro cell assays under hypoxic conditions. The mechanism of CoCl_2_ to induce hypoxic conditions is similar to the hypoxic microenvironment in vivo, which stabilizes the transcription factor HIF-1α [37,38]. Therefore, CoCl_2_ was used to induce hypoxia in this study.

In the present study, we found that the indole-based benzenesulfonamide derivatives at 100 µM significantly inhibited the viability of breast, lung, and pancreatic cancer cells in CoCl_2_-induced hypoxic conditions, with more potent inhibition on breast cancer cell lines than lung or pancreatic cancer cells (Figure 2). According to our preliminary results, compounds **A1** and **A6** on MDA-MB-231 cells and **A6** and **A15** on MCF-7 and SK-BR-3 cells showed higher cytotoxicity than the other compounds, exerting more than 50% inhibition of cell viability. Therefore, compounds **A1**, **A6**, and **A15** were chosen to determine their IC_50_ values on these breast cancer cells. These findings agree with our previous report, in which higher inhibitory activities are observed against the human CA IX isoform by **A1**, **A4**, **A6**, and **A15** [20]. Although **A1**, **A6**, and **A15** exhibited similar cytotoxic patterns in three types of breast cancer cells, the IC_50_ values of **A1** and **A6** on MDA-MB-231 cells were determined to be over 100 µM (Figure 3). By contrast, the calculated IC_50_ values of **A6** and **A15** on MCF-7 and SK-BR-3 cells were close to 50 μM, indicating that these cells were more sensitive than MDA-MB-231 cells to **A6** and **A15**. MDA-MB-231 is a highly aggressive, invasive, and poorly differentiated triple-negative breast cancer cell line, lacking ER and progesterone receptor expression as well as HER2 amplification [39]. Thus, our data showing low sensitivity of MDA-MB-231 cells to our compounds are in parallel with this evidence. While the potency of **A1** to inhibit the viability of MDA-MB-231 cells was inferior to that of SLC-0111, the reference compound known to inhibit CA IX and XII isoforms, **A6** and **A15** exhibited superior inhibition of cell viability than SLC-0111 on MCF-7 and SK-BR-3 cells (Figure 3). Therefore, these compounds (**A6** and **A15**) were selected for further investigation of their cytotoxic effects on MCF-7 and SK-BR-3 cells.

Among the 16 isoforms, CA IX and CA XII have been strongly implicated in cancer [5,40]. CA IX is detectable in several types of human carcinoma cells but not in adjacent healthy tissues, implying its crucial role in cancer [41]. The expression of CA IX was upregulated by CoCl_2_ treatment in both MCF-7 and SK-BR-3 cells (Figure 4). Although several compounds, including our compounds (**A6** and **A15**), have been reported to potently inhibit the human CA II isoform with *Ki* values of around 10 nM, compounds **A4**, **A6**, and **A15** were also shown to inhibit CA IX with *Ki* values of 165.5, 165.1, and 169.6 nM, respectively [20]. Consistent with this report, the present study demonstrates that **A6** and **A15** strongly suppress the CoCl_2_-induced CA IX expression in both cell types (Figure 5). Upon knockdown of the CA IX isoform, the viability of MCF-7 and SK-BR-3 cells was significantly decreased (Figure 6A), indicating that CA IX played a crucial role in the survival of these cancer cells. While **A6** and **A15** treatment significantly suppressed the survival of the control siRNA-transfected cells, these compounds did not affect the viability of cells transfected with CA IX siRNA (Figure 6A), demonstrating that CA IX is required to exert cytotoxic effects. In light of previous evidence that these compounds also inhibit CA II and XII [20], we additionally tested the effects of CA II or XII knockdown with the corresponding siRNAs on the viability of both cell types. While the knockdown of CA II did not affect the viability of MCF-7 and SK-BR-3 cells, CA XII knockdown markedly reduced the viability of these cells (Figure 6B,C). These results demonstrate that, in addition to CA IX, CA XII also plays an important role in tumor cell survival. However, the viability of CA XII-knockdown cells was further reduced by **A6** or **A15** (Figure 6C), indicating that CA XII did not mediate the cytotoxic effects of these compounds on these breast cancer cells. Collectively, these findings corroborate the vital role of CA IX in tumor cell survival and validate the cytotoxic effects of **A6** and **A15** on MCF-7 and SK-BR-3 cells through inhibition of CA IX.

The modulation of pH by CAs can promote tumor cell migration to survive in hypoxic regions and contribute to the metastasis of tumor cells [15]. In MCF-7 cells, **A6** abated the cell migration, while no significant effect was observed with **A15** (Figure 7A). In SK-BR-3 cells, both **A6** and **A15** reduced cell migration, and the antimigratory effects of these compounds were comparable to that of SLC-0111 (Figure 7B). Taken together, our data demonstrate that **A6** and **A15** possess CA IX-mediated antitumor and antimigratory effects on MCF-7 and SK-BR-3 cells.

To date, efforts have been made to chemically modify the structures of sulfonamides to identify specific inhibitors of the CA IX isoform. Several small molecule inhibitors were identified with anticancer activities in xenograft and metastatic animal models and even in a phase Ia clinical trial [16,19]. However, CA IX is also implicated in the development of drug resistance [6,35]. In order to avoid the possible drug resistance and/or to improve its cytotoxic efficacy, attempts have been made to study the effects of CA IX inhibitors in combination with conventional chemotherapy [42,43,44]. For instance, in vitro preclinical studies demonstrated that SLC-0111 complemented and potentiated the cytotoxic effects of conventional chemotherapeutic drugs, including dacarbazine and temozolomide in A375-M6 melanoma cells, DOX in MCF-7 breast cancer cells, and 5-fluorouracil in HCT116 colorectal cancer cells [19]. Given these reports, we also assessed the cytotoxic effects of **A6** and **A15** in combination with a conventional anticancer agent DOX on MCF-7 and SK-BR-3 cells. DOX is widely used to treat cancer, including breast cancer, bladder cancer, and lymphoma, often together with other chemotherapy to enhance its therapeutic efficacy. However, the use of this drug is gradually limited due to its low tumor selectivity, side effects, and drug resistance [45]. In this regard, we selected DOX for our combination treatment study. DOX exerted a potent antitumor effect on MCF-7 and SK-BR-3 cells, with respective IC_50_ values of 0.95 and 0.64 μM (Figure 8). Based on their IC_50_ values determined in this study on MCF-7 and SK-BR-3 cells, we tested the combined effects of **A6** or **A15** with DOX at the concentrations of fixed and non-fixed ratios, as illustrated in Figure 9, and found significantly enhanced cytotoxic effects of **A6** or **A15** in combination with DOX (Figure 10).

We further analyzed the cytotoxic effects of our drug combinations by two different methods, Chou−Talalay and Bliss independence, and demonstrated their synergism. According to the Chou−Talalay method, the combination of **A6** or **A15** with DOX resulted in synergistic effects in almost all pairs of drug combinations on both cell types, with CI values below 1 (Table 1 and Figure 11). Similarly, the Bliss independence model proved the overall synergism in most of the combination pairs tested, except for the combination of **A6** and DOX on MCF-7 cells (Figure 12), providing slightly different results from the Chou−Talalay method. While the Chou−Talalay method presented synergism in most pairs of the combination of **A6** with DOX on MCF-7 cells (Table 1 and Figure 11), the Bliss score indicated an additive effect for this combination (Figure 12). The difference between the two methods may be due to the inclusion of non-fixed-ratio combinations in the Bliss independence model for the synergy evaluation. Among the combinations tested in this study, the combination of **A15** and DOX was the most promising pair to exert synergistic cytotoxicity, particularly on SK-BR-3 cells.

Finally, the effects of **A6** or **A15** in combination with DOX on cell migration were evaluated in SK-BR-3 cells. The cells treated with **A6** or **A15** and DOX for 48 h exhibited enhanced inhibition of cell migration (Figure 13). The enhanced antimigratory effect of the combined treatment with **A15** and DOX was significantly different from that observed with **A15** or DOX alone, exhibiting synergistic inhibition of cell migration. It would be valuable to further explore signaling molecules mediating the antimigratory effect of the combined treatment with **A15** and DOX. For example, modifications of several vital proteins involved in invasion, migration, and metastasis, such as collagens I, II, and IV, fibronectin, laminin, tenascin, and vitronectin, by the combined treatment could be the targets for further investigation [46]. Previous studies suggested that hypoxia could contribute to the development of resistance to DOX because the uptake of this drug might be negatively affected by the extracellular acidification of the hypoxic tumor microenvironment [47]. Therefore, targeting CA IX to control pH may efficiently enhance the delivery of DOX to the hypoxic tumor, thereby improving its therapeutic efficacy and minimizing chemoresistance. Further studies using animal models are required to verify the synergistic anticancer effects of the combined treatment of DOX with **A6** or **A15**. Furthermore, it would be intriguing if we can extend our study in the future to compare the synergistic effects of our combination treatment to the effects of the drug combination of DOX used in clinical situations to treat breast cancer.

## 4. Materials and Methods

### 4.1. Materials and Chemicals

A series of 15 benzenesulfonamide derivatives (**A1**–**A15**) were prepared as described [20]. For all experiments, a stock solution of each test compound was prepared in DMSO at 50 mM concentration and serially diluted to the desired concentrations in the respective culture media.

Dulbecco’s modified Eagle medium (DMEM), Roswell Park Memorial Institute (RPMI), and fetal bovine serum (FBS) were purchased from Corning, Inc. (Corning, NY, USA). DMSO and 3-(4,5-dimethylthiazol-2-yl)-2,5-diphenyltetrazolium bromide (MTT) were bought from Sigma-Aldrich (St. Louis, MO, USA). SLC-0111 was obtained from MedChemExpress (Monmouth Junction, NJ, USA). Anti-CA I (1:1000, cat#ab267475) antibody was purchased from Abcam (Cambridge, MA, USA), and antibodies specifically recognizing CA II (1:1000, cat#8612), CA IX (1:1000, cat#5649), and CA XII (1:1000, cat#5865) were from Cell Signaling Technology (Danvers, MA, USA). CA II, CA IX, and CA XII siRNA and control siRNA were provided by Santa Cruz Biotechnology, Inc. (Dallas, TX, USA).

### 4.2. Cell Culture

All cell lines, including the three human breast cancer cell lines (MDA-MB-231, MCF-7, and SK-BR-3), human pancreatic cancer Panc1 cells, and human A549 lung cancer cells, were obtained from the American Type Culture Collection (ATCC, Manassas, VA, USA). Breast cancer cell lines and Panc1 cells were cultured in DMEM containing 10% FBS and 1% antibiotic–antimycotic (final concentrations of 100 U/mL penicillin, 100 µg/mL streptomycin, and 0.25 µg/mL amphotericin B). A549 lung cancer cells were cultured in RPMI containing 10% FBS and 1% antibiotic–antimycotic (composition given above). All cells were cultured and maintained in a humidified incubator at 37 °C with 5% CO_2_ until 80–90% confluence and then trypsinized with 0.05%/0.5 mM trypsin−EDTA for experimental assays or a new passage culture, as previously described [48,49].

### 4.3. Measurement of Cell Viability

To determine the effects of the synthesized compounds on cell viability, MTT assays were performed as previously described [50]. Briefly, a total of 0.5 × 10^4^ MDA-MB-231, 1 × 10^4^ MCF-7, 2 × 10^4^ SK-BR-3, 2 × 10^4^ Panc1, and 0.5 × 10^4^ A549 cells (100 μL/well) were seeded on the flat bottom of 96-well plates and grown overnight at 37 °C in a 5% CO_2_ atmosphere. Then, the cells were treated with each test compound alone or in combination with DOX for 48 h. For the vehicle-treated control group, sister cultures were treated with the respective media containing DMSO. The final concentrations of DMSO were no more than 0.6%. DMSO concentrations used to treat control cells in each experiment are stated in the corresponding figure legends, and did not exhibit significant cytotoxic effects in the cancer cell lines used in this study. After the desired treatment, the media containing test compounds were carefully removed to minimize any influence of their color on the MTT assay [50]. Then, 100 μL of MTT solution (0.5 mg/mL of working concentration) was added to each well for 3 h. Blue formazan crystals at the bottom of wells were dissolved in DMSO. The absorbance was read at 550 nm using a microplate reader (SpectraMax M2e, Molecular Devices, Sunnyvale, CA, USA). The cell viability was calculated as the percentage of absorbance measured in the vehicle-treated control cells.

The dose−response curves were generated using GraphPad Prism 5 statistical analysis software (GraphPad Software, Inc., La Jolla, CA, USA), and the IC_50_ values were calculated via nonlinear regression using the same software.

### 4.4. Cell Migration Assay

To measure the effects of the test compounds on cell mobility, wound healing assays were conducted as previously reported [50,51]. MCF-7 and SK-BR-3 cells were plated in 24-well plates at a density of 1 × 10^5^ and 1.5 × 10^5^ cells/well, respectively, and incubated overnight at 37 °C until 70–80% confluence. After a wound line was created by a sterile scratcher in each well, the cells were treated with the compounds in serum-free media. Control cells were treated with serum-free media containing DMSO instead. The concentrations of DMSO are provided in the corresponding figure legends. The cell migrations during 24 or 48 h were determined by comparing the area of the wounds at 0 and 24 or 48 h, respectively, using ImageJ software version 1.49. The difference of the wound area at 0 and 24 or 48 h time points measured in the vehicle-treated control cells was considered 100%, and cell migration of the compound-treated cells during 24 or 48 h was expressed as a percentage of the respective control-treated cells [52].

### 4.5. Western Blotting

MCF-7 and SK-BR-3 cells were plated in 35-mm culture dishes at a density of 4 × 10^5^ and 6 × 10^5^ cells/well, respectively. The cells were treated with **A6** and **A15** (50 and 100 µM) for 48 h in the presence or absence of CoCl_2_ (100 µM). The cells were washed and lysed with lysis buffer on ice. Electrophoresis and immunoblotting were performed according to procedures reported previously [49,50]. Briefly, lysates containing equal amounts of proteins were resolved by SDS-PAGE and transferred to nitrocellulose membranes at 100 V for 90 min. Membranes were blocked with 5% skim milk at room temperature for 90 min and incubated with primary antibodies (anti-CA I, II, IX, and XII antibodies) in BSA at 4 °C overnight. After rinsing with Tris-buffered saline containing 0.1% Tween 20, membranes were incubated with HRP-conjugated secondary antibodies at room temperature for 90 min. Finally, blots were visualized with a Bio-Rad ChemiDoc XRS imaging system using enhanced chemiluminescence reagents (Bio-Rad, Hercules, CA, USA).

### 4.6. Transfection with siRNA

MCF-7 and SK-BR-3 cells were plated in 60-mm culture dishes at a density of 1 × 10^6^ and 1.5 × 10^6^ cells/well, respectively, and incubated at 37 °C overnight to 75–80% confluence [49]. Afterward, control siRNA and siRNA targeting CA II, IX, or XII were transfected using Lipofectamine 3000 transfection reagent (Invitrogen, Rockford, IL, USA), according to the manufacturer’s instructions. After 6–8 h of transfection, media containing siRNAs were replaced with new culture media, and cells were seeded for further experiments, including western blotting and MTT assay.

### 4.7. Analysis of Drug Combination Effects

To quantify the synergistic effect of the test compounds (**A6** and **A15**) in combination with DOX, we first estimated the combination indices (CIs) by the Chou−Talalay method using CompuSyn software (ComboSyn, Inc., Paramus, NY, USA), as described at https://www.combosyn.com/ (accessed on 10 May 2022) and in a previous report [31]. The two drugs were combined in fixed ratios of concentration corresponding to 0.25-, 0.5-, 1-, 2-, and 4-fold the individual IC_50_ values. The CI is a quantitative value indicating the synergism of a drug combination at a specific concentration calculated by the formula previously described [46]. The CI values less than 1, equal to 1, and more than 1 indicate synergism, additivity, and antagonism, respectively. The lower the CI, the stronger the synergism.

The synergism was also evaluated by the Bliss independence model using SynergyFinder at https://synergyfinder.fimm.fi (accessed on 10 May 2022). The two drugs were combined in the fixed and non-fixed ratios of concentrations, as indicated in Figure 9. Synergy scores below −10, from −10 to 10, and above 10 indicate antagonism, additivity, and synergism, respectively.

### 4.8. Statistical Analysis

All data were presented as the mean ± SEM from at least three independent experiments in triplicates. Comparisons were tested by one-way analysis of variance (ANOVA) followed by Tukey’s post hoc test using SigmaPlot 12.5 (Systat Software, San Jose, CA, USA). A *p*-value < 0.05 was considered statistically significant.

## 5. Conclusions

In conclusion, novel CA inhibitors possessing indole-based benzenesulfonamide moieties were evaluated for antitumor and antimigratory effects on various cancer cell lines, including breast, lung, and pancreatic cancer cells. Among the 15 analogs tested, **A6** and **A15** exhibited potent cytotoxic and antimigratory activities against MCF-7 and SK-BR-3 breast cancer cells in the CoCl_2_-induced hypoxic conditions. Using CA IX-knockdown cells, we demonstrated that **A6** and **A15** exerted cytotoxic effects through the inhibition of CA IX. Although these compounds were recently reported to potently inhibit human CA II and XII, the knockdown of these isoforms did not affect the **A6**- or **A15**-induced cytotoxicity on both cell types. Therefore, these isoforms are unlikely to participate in the antitumor activities of our compounds. Based on analyses using Chou−Talalay and Bliss independence methods, **A6** and **A15** in combination with DOX exerted synergistic anticancer and antimigratory effects on these breast cancer cells. Among the tested pairs, the combination of DOX with **A15** showed the strongest synergism on SK-BR-3 cells. Taken together, the present study demonstrated that compounds **A6** and **A15** suppressed tumor cell survival and migration of MCF-7 and SK-BR-3 cells through inhibition of CA IX. Moreover, the combination of these compounds with DOX exhibited synergistic cytotoxic and antimigratory effects on these breast cancer cells. Based on our findings, **A6** and **A15** may serve as potential anticancer agents used alone or in combination with DOX against breast cancer. Future studies are warranted to confirm our findings in vivo and to further characterize optimal combinations for maximal efficacy.

## Figures and Tables

**Table 1:** Fractional effects (*F_a_*) and combination index (CI) values of the combination treatment of doxorubicin (DOX) with **A6** or **A15** at fixed ratios on MCF-7 and SK-BR-3 cells.

Cell Type	Combination	Concentration (μM)		*F_a_*	CI
		DOX	A6 or A15		
MCF-7 cells	DOX + **A6**	0.25	12.5	0.29013	0.76587
		0.5	25	0.43179	0.80505
		1.0	50	0.56273	0.91602
		2.0	100	0.71379	0.93939
		4.0	200	0.80495	1.11489
	DOX + **A15**	0.25	12.5	0.51398	0.26533
		0.5	25	0.66158	0.19980
		1.0	50	0.66083	0.39441
		2.0	100	0.79236	0.33441
		4.0	200	0.90957	0.20999
SK-BR-3 cells	DOX + **A6**	0.125	12.5	0.42113	0.53439
		0.25	25	0.46859	0.78430
		0.5	50	0.60618	0.71892
		1.0	100	0.63780	1.22094
		2.0	200	0.90300	0.52618
	DOX + **A15**	0.125	12.5	0.45368	0.35666
		0.25	25	0.66849	0.21808
		0.5	50	0.62930	0.54761
		1.0	100	0.74096	0.55151
		2.0	200	0.87966	0.16656

Cytotoxic effects of the combined treatments of DOX with **A6** or **A15** at fixed ratios were determined by MTT assay in MCF-7 and SK-BR-3 cells and analyzed by Chou−Talalay method using CompuSyn software, as described in the Materials and Methods. CIs below 1 (highlighted in red) represent synergism.

## Data Availability

Not applicable.
